# IGF1R VARIANT R407H PREVENTS COGNITIVE IMPAIRMENT AND NEUROINFLAMMATION IN CRISPR MICE WITH AAV AB42 AND PTAU

**DOI:** 10.1093/geroni/igae098.3665

**Published:** 2024-12-31

**Authors:** Joo Young Park, Jackson Wezeman, Ruby Sue Mangalindan, Warren Ladiges

**Affiliations:** University of Washington, Seattle, Washington, United States; University of Washington, Seattle, Washington, United States; University of Washington, Seattle, Washington, United States; University of Washington, University of Washington, Washington, United States

## Abstract

Late-onset Alzheimer’s disease (AD) is an age-related disease. The geroscience concept dictates that interventions that slow aging should also slow age-related diseases. This concept was tested in C57BL/6 mice expressing a CRISPr engineered IGF1R variant R407H shown to be enriched in centenarians. A total of 14 females, 25 to 28 months of age, carrying the R407H variant or wild type, were given intravenous injections of AAV9-PHP.eb serotype concoctions at 10^12 vg per dose, containing either a mCherry-sham construct or Aβ42 and pTau constructs associated with neuropathology of AD. Mice were followed for 11 weeks and then tested for cognition. Following euthanasia, brains were collected, and hippocampal tissues processed for RNA sequencing (Novogene, Sacramento). The mice with the IGF1r variant, and infected with AAV Aβ42 and pTau, showed significantly better performance in a spatial navigation learning task compared to wild type cohorts. RNA sequencing analysis in the same cohorts using DES Seq2 showed a significant downregulation of inflammatory cytokines and chemokines including Ccl21b (log2 fold change = -2.3, adjusted p-value < 0.05). These preliminary observations suggest the potential of further studying naturally occurring rare variants such as IGF1r R407H as a way to help slow or prevent AD, in line with the geroscience concept.

